# Direct amplitude-only hologram realized by broken symmetry

**DOI:** 10.1126/sciadv.adp1205

**Published:** 2024-08-30

**Authors:** Daeho Yang, Hong-Seok Lee

**Affiliations:** ^1^ Department of Physics, Gachon University, Seongnam, Gyeonggi-do, South Korea.; ^2^ Major of Electrical Engineering, College of Engineering, Pukyong National University, Busan, South Korea.

## Abstract

Holographic displays have been a long-standing ambition for decades to realize true-to-life reconstruction. However, their practical adoption is hindered by their subpar image quality compared to two-dimensional displays, which is fundamentally limited by restricted spatial frequency bandwidth and artifacts. We address the limitation by using a symmetry-broken amplitude-only spatial light modulator, demonstrating image quality comparable to that of two-dimensional displays. The broken conjugate symmetry induced by phase noise of modulators eliminates conjugate image that causes issues in amplitude-only holograms and allows direct reconstruction without additional optical elements. The proposed method provides enhanced robustness against artifacts caused by sub-pixel structures of modulators, enabling experimental reconstruction of high-quality holograms. The full bandwidth and the robustness result in a 5-decibel improvement in peak signal-to-noise ratio compared to state-of-the-art holograms. Furthermore, the hologram has 24 times higher optical efficiency and a smaller volume than the traditional amplitude-only holograms while real-time synthesis is enabled by using a neural network.

## Teaser

Phase noise in modulators enables high-quality holograms with improved efficiency, a simpler system, and real-time synthesis.

## INTRODUCTION

Holography refers to the process of recording and reconstructing the wavefield of an object by using diverse techniques including interference between fields (*1*), self- interference (*2*), and interferenceless methods using coded aperture (*3*). Because holography can directly recover phase information from records, it has been adopted in various applications, including biomedicine (*4*), data storage (*5*), and nondestructive testing (*6*). However, the recorded data reflect only the intensity of the interference pattern, not the wavefield, so the phase-conjugate wavefield of the object field is also recorded (*7*). As a result, twin images are produced during the playback of the recorded wavefield, hindering accurate recovery (*8*), and, thus, several studies including four-step phase-shifting technique synthesized twin-image–free hologram.

Phase-conjugate symmetry also exists in the reconstruction process of holographic displays, and the symmetry produces a conjugate image (*9*), a physically equivalent phenomenon of a twin image (*10*). The conjugate image appears on the opposite side of the target image, misleading the perception of the three-dimensional scene and producing substantial noise (*9*, *11*). To suppress the conjugate image, the hologram is converted to a complex field by lifting the degeneracy between the conjugate and object images and blocking the conjugate image using an optical filter (*12*, *13*). However, optical filtering also blocks high-frequency regions in the Fourier domain, so complex field conversion reduces the spatial frequency bandwidth of the hologram (*12*, *14*), resulting in blurriness of the reconstructed image.

Given that most photorealistic hologram synthesis methods use complex field conversion in conjunction with optical filtering (*15*–*20*), the reduced frequency bandwidth problem is inseparable from the holograms. Superpixel methods, for example, combine several pixels of a spatial light modulator (SLM) to represent a complex value and eliminate irrelevant pixel information through filtering (*16*, *17*). Double-phase encoding methods (*15*, *20*) and hologram bleaching methods (*18*, *19*) isolate the desired wavefield from the noisy field by applying periodic phase and screening noise frequencies. Recent studies have proposed methods for depicting more details containing high frequencies, along with the synthesization of noiseless holograms (*21*, *22*). However, because the amount of detail in the reconstructed hologram is proportional to the maximum frequencies that can be expressed, the reduced spatial frequency bandwidth inherently constrains image quality.

On the contrary, holograms can be synthesized by using intensity-only reconstruction (*23*), in which only the intensity of an object is set as the reconstruction target instead of the entire complex wavefield. In intensity-only reconstruction, the full bandwidth of the SLMs can be effectively used due to the absence of filters. However, unblocked higher orders of diffraction produced by sub-pixel structures of SLMs cause substantial artifacts, and their image quality is far lower than complex-converted holograms (*24*–*27*). Various approaches including incorporation of sub-pixel structure considerations in a phase-only SLM (*26*) and optimization of temporal multiplexing in an amplitude-only SLM (*28*) developed to reduce artifacts, but the demonstrated image quality is still lower than that of complex holograms.

Here, we propose a direct amplitude-only hologram (DAOH), which offers higher image quality than conventional holograms by using the full spatial frequency bandwidth of an SLM. Furthermore, we present that the DAOH is more robust against sub-pixel artifacts due to its smooth phase, and, thus, enhanced image quality can be realized in the experiment. The phase noise of the SLM transforms the modulated signal from a symmetric straight line to an asymmetric curve on the Argand plane and breaks conjugate symmetry. By confining the wavefield values to the curve, the hologram can be directly represented on the SLM, resulting reconstruction of the hologram without a conjugate image. Because the hologram can be directly reconstructed without optical filters (*25*) or other components (*24*, *26*, *29*), DAOH overcomes the main disadvantage of amplitude-only holograms, i.e., low optical efficiency, and offers a smaller volume. The three major advantages of DAOH—great image quality, high efficiency, and small volume—are desired factors for compact holographic displays, and, thus, DAOH has sufficient potential to be used in a variety of applications including augmented and virtual realities. Moreover, the robustness of DAOH against sub-pixel artifacts can serve as inspiration and be applied to other algorithms.

## RESULTS

For a wavefield at the *z* = 0 plane, *U*(*x*, *y*, *z* = 0), we define the DAOH without phase noise as the wavefield satisfying the following equations

**Fig. 1.**
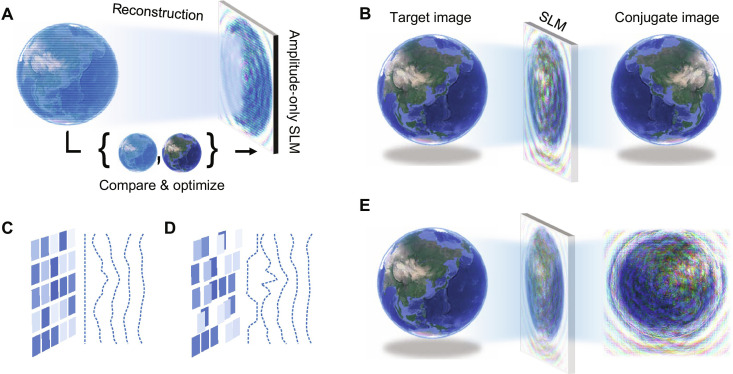
Schematics of DAOH. (**A**) Intensity-only reconstruction using an amplitude-only SLM. (**B**) Reconstructed target image and conjugate image of an amplitude-only hologram without phase noise. (**C** and **D**) Equiphase plane with light intensity modulated by an SLM without phase noise (C) and with phase noise (D). Because of the additional phase modulation, the equiphase plane at the SLM is no longer a flat plane and affects the propagation of light. (**E**) Reconstructed target image of DAOH without a conjugate image.

U(x,y,z=0)=|U(x,y,z=0)| (1)

$$\left|U(x,y,z=d){|}^{2}=I_{\text{target}}(x,y\right)$$ (2)

where *U*(*x*, *y*, *z* = *d*) is the propagated wavefield for a distance *d* along the *z* axis and *I*_target_(*x*, *y*) is the target intensity. While anchoring the amplitude of the wavefield at the object plane as the target amplitude, modulating the phase at the object plane can alter the phase at the SLM plane, and it is feasible to reduce the phase variation of the field at the SLM plane. As the phase variation approaches zero, the field can be considered as a zero-phase field with a global phase (Eq. 1), and the entire wavefield can be directly reconstructed by simply displaying the intensity, |*U*(*x*, *y*, *z* = 0)|^2^, on an ideal amplitude-only SLM (Fig. 1A). Moreover, the propagated intensity reconstructs the target intensity if the wavefield satisfies Eq. 2. While intensity-only reconstruction has been widely studied in phase-only SLMs (*26*, *30*, *31*), intensity-only reconstruction in amplitude-only SLMs without artifacts has not yet been reported.

The dilemma of the amplitude-only representation is that the hologram reconstructs the conjugate image at the *z* = −*d* plane as well as the target image at the *z* =*d* plane (Fig. 1B). By using Eq. 1 and the Fresnel approximation, we can simply derive the relation (see Materials and Methods)

$$U(x,y,z=-d)=U^{*}(x,y,z=d)$$ (3)

Equations 2 and 3 imply that the twin image is reconstructed at *z* = −*d* plane by the condition |*U*(*x*, *y*, *z* = −*d*)|^2^ = |*U*(*x*, *y*, *z* = *d*)|^2^ = *I*_target_(*x*, *y*), and, consequently, the twin image of DAOH is inevitable.

However, a few types of SLMs, including liquid crystal-based devices, do not work ideally; therefore, not only the amplitude of the field is modulated, but also the phase of the field is modulated (*32*). Owing to the additional phase modulation, the equiphase plane of the reconstructed field in front of the SLM is curved (Fig. 1D), breaking symmetry of the propagated wavefield contrary to the flat plane of the ideal SLM (Fig. 1C). By assuming additional phase modulation as an arbitrary function of the intensity, ϕ(|*U*(*x*, *y*, *z* = 0)|^2^), Eq. 1 should be modified to directly reconstruct holograms, and the modified condition is given as

$$U(x,y,z=0)=e^{i\phi \left({\left|U\left(x,y,z=0\right)\right|}^{2}\right)}|U(x,y,z=0)|$$ (4)

Although unwanted phase generally disturbs reconstruction of holograms, consideration of the anticipated phase modulation during the hologram synthesis can eliminate the twin image and prevent wavefield degradation by the phase noise (Fig. 1E). The broken symmetry also leads to an asymmetric defocus blur; the defocus blur on one side is much stronger than that on the other side (see the Supplementary Materials for further details).

The solution satisfying Eqs. 2 and 4 can be obtained by optimizing the wavefield using a gradient descent method. The optimization begins by calculating the absolute values of an arbitrary wavefield *U*(*x*, *y*) and applying the phase noise of the SLM to the field. The wavefield affected by the noise propagates for a distance *z*, where the propagation is modeled by the angular spectrum method (ASM) (*33*). The intensity of the propagated wavefield is compared with the target intensity, and the loss function is calculated from the difference. In summary, the total loss function, *ℒ*, is expressed as

$$ℒ=\frac{1}{N}\sum_{x,y}‍{\left[{\left|Fr_{d}\left\{e^{i\phi \left({\left|U\right|}_{\text{clip}}^{2}\right)}{\left|U\right|}_{\text{clip}}\right\}\right|}^{2}-I_{\text{target}}(x,y)\right]}^{2}$$ (5)

where |*U*|_clip_ ≡ min {max{|*U*(*x*, *y*, *z* = 0)|,0},1}, Fr*_d_*{⋯} is the propagation operator, *N* is the number of pixels, and *I*_target_(*x*, *y*) is the target intensity. Although we adopted the gradient descent method to synthesize DAOH, the large computational cost can be reduced by adopting neural networks without degrading image quality. Image quality and synthesis speed benchmarks of the neural encoding method are presented in the Supplementary Materials.

**Fig. 2.**
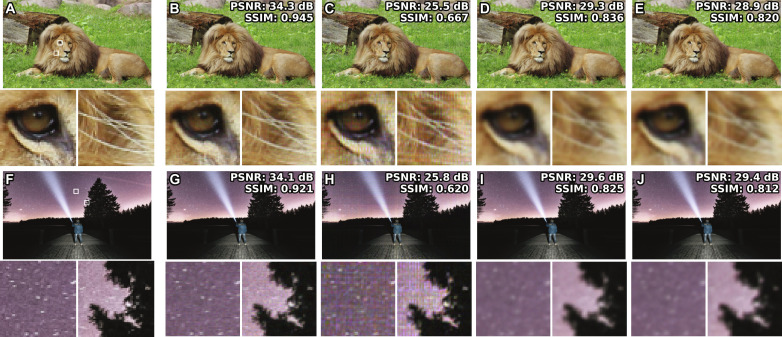
Numerically reconstructed intensities of holograms. Reconstruction target images (**A** and **F**) and enlarged images of them are presented, while the enlarged area is marked as white boxes. Reconstructed intensities of DAOHs (**B** and **G**), phase-only holograms (**C** and **H**), complex holograms with double-phase encoding (**D** and **I**), and complex holograms with Burch encoding (**E** and **J**) at the target plane. Peak signal-to-noise ratios (PSNRs) and structural similarity index measures (SSIMs) of reconstructed intensities compared to the target intensities are marked at the top right side of the images. For DAOHs and complex holograms (phase-only holograms), the distances between the target and SLM planes were set to 2 (250) mm. Because the phase-only holograms have uniform intensity at the SLM plane, reconstructed intensities with a 2-mm propagation distance were noisy white images and thus we used a 250-mm propagation distance in the phase-only holograms. The pixel pitch is assumed to be 7.2 μm, and the pixel size is assumed to be 7.0 μm, corresponding to the fill factor of 0.95. Numerical reconstruction is modeled by ASM.

The numerically reconstructed intensities of a DAOH, the phase-only hologram based on the stochastic gradient descent (SGD) algorithm (*34*), the complex hologram encoded by the anti-aliasing double phase method (*20*), and the complex hologram encoded by the Burch method (*14*, *35*) are presented in Fig. 2. The phase-only hologram refers to the intensity-only reconstruction using a phase-only SLM. To numerically reconstruct the holograms, we adopted the ASM method, considering the sub-pixel structures of SLMs (see Materials and Methods). Because the sub-pixel structures include the fine details of the pixels and the black matrix, the higher-order terms of the SLM can be effectively considered. Among the holograms, DAOH presents the best image quality, even better than those of the complex holograms. In the case of the complex holograms, optical filtering is required to convert a constant-amplitude or constant-phase field into a complex field, and the blocked high-frequency components inhibit the utilization of the full resolution of the SLM. To avoid noise originating from high frequency cutoff, Gaussian blur is applied to the images before synthesizing complex holograms (*20*), whereas the variance of the Gaussian blur is tuned using the numerically reconstructed peak signal-to-noise ratio (PSNR). It should be noted that we selected images having more high-frequency components to enhance the visibility of the differences. In cases with fewer high-frequency components, the differences may be less substantial than those presented. A comparison with low-frequency images can be found in the Supplementary Materials.

Photo credit: Appaloosa

Ideally, intensity-only reconstruction should produce better image quality than complex holograms owing to the absence of spatial frequency bandwidth limitations. However, the image quality of the phase-only holograms based on the SGD algorithm is degraded by the sub-pixel structure, resulting in intensity disparities between propagations with and without considering the sub-pixel structure. In contrast, the sub-pixel structure noise can be mitigated in amplitude-only holograms. It is possible to explain that the noise of the sub-pixel structure is proportional to the phase gradient in the first-order approximation using the transport-of-intensity equation (TIE; see Materials and Methods). In other words, the smooth phase of holograms reduces artifacts, as reported in other studies (*36*–*39*).

Although smooth phase holograms can be reconstructed using phase-only SLMs, it is noteworthy that implementing smooth phase holograms using amplitude-only SLMs is more straightforward, particularly without optical filters. For instance, phase-only SLMs without optical filters struggle to maintain a smooth phase due to the substantial intensity variations from the SLM plane to the target plane (*26*). On the other hand, amplitude-only SLMs naturally foster a smooth phase by directly adjusting intensity at the SLM plane. As a result, the image quality of amplitude-only holograms is not substantially degraded even without an optical filter. We avoid limiting bandwidth in intensity-only reconstructions to preserve their distinctiveness, as optical filtering not only reduces artifacts but also diminishes image sharpness, similar to complex holograms.

**Fig. 3.**
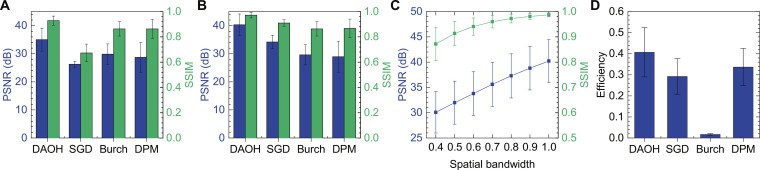
Image quality benchmark of the holograms. (**A**) PSNRs and SSIMs of the holograms reconstructed by the numerical method considering the sub-pixel structure. The pixel pitch is assumed to be 7.2 μm, and the pixel size is assumed to be 7.0 μm. DPM refers to the double-phase encoding method, and Burch refers to the Burch encoding method. (**B**) PSNRs and SSIMs of the holograms reconstructed by the numerical method without considering the sub-pixel structure. (**C**) PSNRs and SSIMs of ideal complex holograms depending on the spatial frequency bandwidth. (**D**) Optical efficiencies of the holograms. The optical efficiencies represent the summation of the numerically reconstructed intensities when the uniform light with a total intensity of 1 is shone on the SLM. In the benchmarks, all images were adjusted to a size of 1920 × 1080 including a gray padding. For the phase-only holograms (other holograms), the propagation distance was set to 250 mm (2 mm) and 160 × 100 (20 × 20) padding was applied (*34*). DIV2K training dataset (*40*) was used as target images of the holograms. The error bar represents the SD between the holograms with different target images.

To concisely compare image quality between holograms, we evaluated image quality metrics using the DIVerse 2K resolution high quality images (DIV2K) dataset (*40*). Figure 3A presents PSNRs and structural similarity index measures (SSIMs) of the holograms when the DIV2K images are adjusted to a size of 1920 × 1080, including gray padding. To demonstrate robustness of DAOH in the presence of sub-pixel structures, we present image quality of the two different cases: one with considering the sub-pixel structure during the numerical reconstruction (Fig. 3A) and another without considering it (Fig. 3B). The second numerical propagation method is the same as the propagation during the hologram synthesis, and the phase-only holograms present great image quality with this numerical reconstruction. However, image quality of the phase-only holograms is substantially reduced when the sub-pixel structure is considered, in contrast to that of the DAOHs. In case of the complex holograms, image quality decrement is negligible because of optical filtering. The image quality differences between the DAOH and the other holograms are greater than 5 dB in PSNR and 0.06 in SSIM. Because PSNR is based on a logarithmic scale, a 5-dB difference indicates that the noise in the DAOH is approximately one-third of that in the other holograms.

In the phase-only holograms based on SGD, the sub-pixel noise is composed of high frequency elements, so elongated propagation distances can eliminate the sub-pixel noise in sake of the blurriness due to the decreased numerical aperture. To reconstruct a sharply focused spot, the wavefield of the point spread function (PSF) should be implemented in the hologram. Because the radius of the PSF is proportional to the propagation distance, sharply focused spot cannot be reconstructed if the radius of the PSF is larger than the SLM. In other words, the spatial frequency bandwidth is restricted by the numerical aperture of the hologram. Detailed information on the image quality depending on the propagation distance can be found in the Supplementary Materials.

The effect of the spatial frequency bandwidth on image quality can be estimated by limiting the spatial frequency bandwidth of an ideal complex hologram. Figure 3C presents the image quality of an ideal complex hologram depending on the spatial frequency bandwidth. The spatial frequency bandwidth is controlled by blocking the high-frequency region in the frequency domain. Although the PSNR can exceed 40 dB when the spatial frequency bandwidth is not limited, a small bandwidth decrease substantially reduces the PSNR. Because the PSNR (SSIM) of the hologram with 0.5 spatial frequency bandwidth is 32.0 dB (0.914), the complex hologram encoded by the double-phase and Burch encoding method cannot overcome this limitation. In contrast, the image quality of DAOHs exceeds the limitation by reconstructing full resolution images.

For the holograms adopting complex field conversion, a major drawback of using amplitude-only SLMs is their low optical efficiency. In contrast to holograms using phase-only SLMs, the optical efficiency is considerably reduced during the conversion from amplitude-only field to complex field. For instance, the optical efficiency of the Burch encoding method, which is prevalent in holograms using amplitude-only SLMs, is less than 2% (*41*). Figure 3D shows the optical efficiencies of the holograms. In DAOH, because the hologram is not optically filtered, the optical efficiency is ~40%, 24 times higher than that of the Burch encoding method. Moreover, the efficiency of DAOH is even higher than that of the phase-only holograms and the double-phase encoding method, which are commonly used methods in phase-only SLMs. The high optical efficiency, combined with high image quality and the absence of optical filtering, makes the system more compact and simpler to implement, making it highly suitable for use in compact devices.

**Fig. 4.**
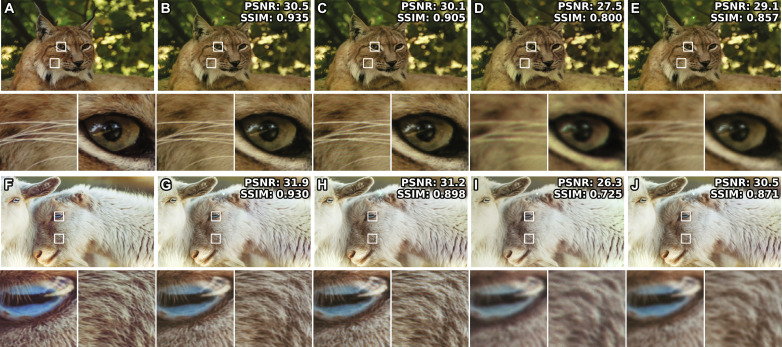
Experimental results of the holograms. Target images (**A** and **F**), experimental setup–limited images (**B** and **G**), optically reconstructed images of DAOHs (**C** and **H**), complex holograms with Burch encoding (**D** and **I**), and spatial frequency bandwidth–limited 2D images (**E** and **J**). To estimate the maximum image quality of our experimental setup, we captured the screen of a 2D display with focusing the camera on the screen (B and G). Image quality metrics compared to the target images are marked on the top right side of the images. Small images present enlarged images of the above images. In the experiments, 1920 × 1080 resolution images were used.

To demonstrate the validity of DAOH explicitly, we conducted an experimental reconstruction of the holograms. Figure 4 shows the experimental results for the DAOHs and complex holograms. The complex holograms were implemented on the amplitude-only SLM by the Burch encoding method, and their frequency bandwidth was reduced to avoid noise originating from high-frequency components (*20*). Because our experimental setup can have imperfections, including optical aberration and SLM-camera pixel mismatch, we captured the SLM displaying two-dimensional (2D) images while the camera focus is at the SLM. The captured images represent the best images of our experimental setup and the image quality metrics of the DAOHs approach this limit, experimentally supporting the theoretical performance of the DAOHs.

Photo credit: Ingo

In contrast, the reconstructed images of the Burch encoding method cannot illustrate the details of the images because of the limited spatial frequency bandwidth. The origin of the degradation in the Burch encoding method can be confirmed by capturing spatial frequency bandwidth–limited 2D images while the camera focus is at the SLM. In the spatial frequency bandwidth–limited 2D images, image quality metrics are slightly higher than those of Burch encoding method, but the blurriness in reconstructed images is similar to that of the Burch encoding method.

It is important to note that the experimental comparison of DAOH and the Burch encoding method does not conclusively establish experimental superiority over other state-of-the-art phase-only holograms. Optical reconstruction results are influenced not only by algorithms but also by experimental apparatus and imperfections such as flicker of SLMs (*42*), types of light sources (*43*), and calibration precisions. Thus, experimental comparison of amplitude-only and phase-only holograms under the same conditions is challenging, and Fig. 4 only demonstrates that the optical reconstruction results of DAOH are minimally affected by the spatial bandwidth, similar to the numerical reconstruction results.

## DISCUSSION

In terms of the total volume of the system, quantitative analysis is a complex task, as it involves considering various factors such as optical aberrations and the number of optical elements. However, if the other conditions are similar, DAOHs can be reconstructed with a smaller system compared to the complex holograms because optical filtering is not necessary (fig. S9). Compared to the phase-only holograms, which require at least tens of millimeter optical path to sufficiently diffract light (*26*), the DAOH does not require a minimum distance between reconstructed holograms and an SLM. Furthermore, employment of a slim-laser backlight can substantially reduce the volume of the combined module of the light source and SLM (*44*), every necessary component of the DAOH, and, thus, the total volume of the system can be much smaller.

**Fig. 5.**
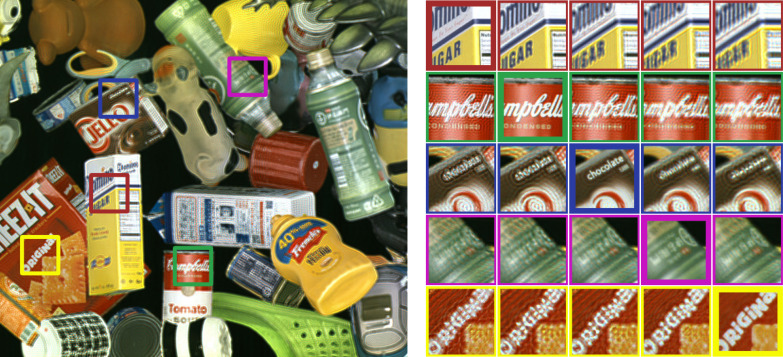
Experimental demonstration of multi-depth DAOH. The large image presents the whole reconstructed intensity of the multi-depth DAOH. The small images present enlarged intensities at different depths and each depth corresponds to 0, 1, 2, 3, and 4 mm (from left to right). The in-focus intensities are marked with thick color boundaries, and the out-of-focus intensities are marked with thin color boundaries.

Although single-plane reconstruction was the main focus of this study, a multi-depth DAOH can be synthesized by optimizing the reconstructed intensity at multiple depths (Fig. 5). A pixel-wise depth mask is applied to the loss function and the loss function is calculated for each depth (*20*, *35*) to synthesize a multi-depth hologram. Moreover, the large computational cost of optimizing the wavefield can be reduced by adopting neural networks (*20*, *34*, *35*), and the results of the proof-of-concept network can be found in the Supplementary Materials. In addition, the successful compensation of the phase noise through amplitude modulation implies that DAOH can realize arbitrary phase modulation and aberration correction as phase-only holograms.

Photo credit: Daeho Yang

Although the smooth phase of the hologram induces robustness in DAOHs, it also has drawbacks. Because of the smooth phase, light scattering of the hologram is restricted. This restricted scattering limits the eyebox to a small size and makes defocus blur weaker (*36*–*39*). While overcoming the limitation of a small eyebox size is challenging, the issue of weak defocus blur can be mitigated by adopting various methods (*28*, *35*). For instance, by adopting an additional loss term, defocus blur can be slightly enhanced in DAOHs (see the Supplementary Materials for further details).

Various hologram synthesis algorithms have been developed recently, but most of the algorithms cannot fully use the spatial frequency bandwidth of SLMs. Although the reduced spatial frequency bandwidth may not be a major concern in laboratory experiments that use large SLMs, it is a decisive factor in compact devices. Because of the limited dots per inch of SLMs, the total volume of the system can be substantially reduced by the capability to express the full bandwidth of the SLMs, as well as the simple optical system of DAOH. Furthermore, high optical efficiency, which is also one of the major challenges of compact devices, can be simultaneously achieved by the direct representation of the hologram. We expect DAOH to be widely adopted in holographic displays including virtual and augmented reality devices, offering the essential aspects of compact displays.

## MATERIALS AND METHODS

### Conjugate symmetry of DAOH

From the Fresnel approximation (*45*), the wavefield at the *z* = *d* plane is given by

$$U\left(x\prime ,y\prime ,z=d\right)=\frac{1}{-i\lambda d}e^{-\mathrm{ikd}}\times \int ‍U\left(x,y,z=0\right)e^{\frac{\pi \left({\left(x-x^{\prime }\right)}^{2}+{\left(y-y^{\prime }\right)}^{2}\right)}{i\lambda d}}\text{dxdy}$$ (6)

By assuming that the wavefield is the DAOH without additional phase modulation, Eq. 6 can be rewritten using Eq. 1 and it is given as

$$\begin{array}{c} U(x\prime ,y\prime ,z=d)\\ \;\;\;=\frac{1}{-i\lambda d}e^{-\mathrm{ikd}}\int ‍\left|U\right|e^{\frac{\pi \left({\left(x-x\prime \right)}^{2}+{\left(y-y\prime \right)}^{2}\right)}{i\lambda d}}\text{dxdy}\\ \;\;\;=\frac{1}{i\lambda \left(-d\right)}e^{\mathrm{ik}\left(-d\right)}\int ‍\left|U\right|e^{\frac{\pi \left({\left(x-x\prime \right)}^{2}+{\left(y-y\prime \right)}^{2}\right)}{-i\lambda \left(-d\right)}}\text{dxdy}\\ \;\;\;=U{\left(x\prime ,y\prime ,z=-d\right)}^{*} \end{array}$$ (7)

Equation 7 tells us that the conjugate symmetry of DAOH holds if there is no additional phase modulation. Although the conjugate symmetry is derived under the Fresnel approximation, it should be noted that the theory is valid for all commercial SLMs due to the small diffraction angles of the SLMs.

### Method to numerically reconstruct hologram

**Fig. 6.**
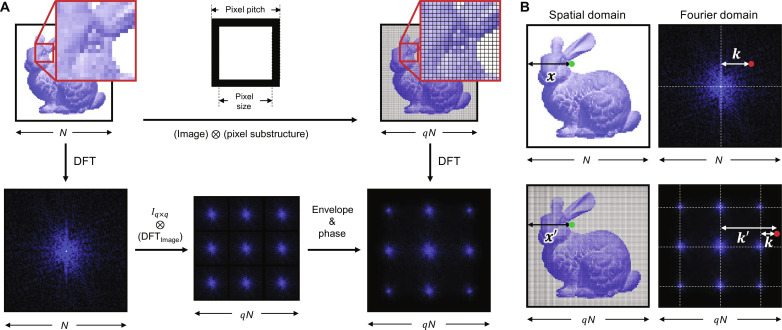
Consideration of sub-pixel structure. (**A**) Two different methods to numerically calculate a sub-pixel structure of an SLM. The first method uses Kronecker tensor product of the whole display and the substructure of one pixel. The second method uses DFT to consider an arbitrary substructure with relatively small memory. We can cross-check the validity of the second method using the first method. (**B**) Relation between the coordinate of the original image and that of the Kronecker tensor product image.

To numerically reconstruct the holograms, we considered the sub-pixel structure of SLMs. A typical liquid crystal on silicon (LCoS) has a fixed fill factor, and the remaining area is filled with black matrix. We emulated such a condition by expanding the size of the hologram by *q* times and putting the same pixel values to the nearest *q* × *q* pixels except for the few pixels, emulating black matrix areas (Fig. 6A). For the few pixels emulating black matrix areas, the values are fixed to zero.

However, *q* times larger matrix calculation is computationally challenging to emulate fill factor larger than 90%, so we used approximate sub-pixel consideration during the numerical reconstruction. After analytically deriving the relation between the hologram in the Fourier domain and the substructure-considered hologram in Fourier domain, we used the formula to calculate sub-pixel–considered wave propagation. The derived formula includes distinct phase factors, compared to the ones reported in the previous study (*26*), because of the utilization of discrete Fourier transform (DFT).

In DFT, the phase factors depend on the number of input values, so different phases are applied to *N* pixels and *qN* pixels during the DFT of the wavefield. From the definition of the DFT, we could calculate the DFT of the wavefield without considering the sub-pixel structure as

$$F_{k}\equiv \text{DFT}(x_{n})=\sum_{n=0}^{N-1}‍x_{n}e^{-i2\pi \mathrm{kn}/N}$$ (8)

Here, *N* is the number of pixels, *x_n_* is the *n*th value in the spatial domain, and *F_k_* is the *k*th value in the Fourier domain.

On the contrary, the DFT of the wavefield considering the sub-pixel structure can be calculated as $F_{k\prime }^{\prime }\equiv \text{DFT}\left(x\prime_{n}\right)=\sum_{n=0}^{N^{\prime }-1}‍x\prime_{n}e^{-i2\pi k^{\prime }n/N^{\prime }}$. To calculate the relation between *F*′_*k*′_ and *F_k_*, we assume that the pixel has no substructure, i.e., fill factor 100%. In that case, we can use the relations, *x_n_* = *x*′*_qn_* = *x*′_*qn*+1_ = ⋯ = *x*′_*q*(*n*+1)−1_ and *N*′ = *qN* (Fig. 6B). Using the relations, we can induce the following equation

$$\begin{array}{cc} F\prime_{k\prime } & =\sum_{n=0}^{N-1}‍\sum_{m=0}^{q-1}‍\left[x_{n}e^{-i2\pi k^{\prime }(\mathrm{qn}+m)/\mathrm{qN}}\right]\\ & =ℰ\left(q,k^{\prime }\right)e^{-i2\pi k^{\prime }\frac{q-1}{2\mathrm{qN}}}\sum_{n=0}^{N-1}‍x_{n}e^{-i2\pi k^{\prime }n/N} \end{array}$$ (9)

Here, $ℰ\left(q,k\prime \right)=e^{i2\pi k^{\prime }\frac{q-1}{2\mathrm{qN}}}\sum_{m=0}^{q-1}‍e^{-i2\pi k^{\prime }m/\mathrm{qN}}$ is an envelope function and goes to *q* · sinc(π*k*′/*N*) when *q* goes to infinity.

By assuming *k*′ = *Nr* + *k*, where *r* is an integer from 0 to *q −* 1, we can induced the relation between *F_k_* and *F*′_*k*′_ as

$$F\prime_{k\prime }=ℰ\left(q,\mathrm{Nr}+k\right)e^{-i2\pi \left(\mathrm{Nr}+k\right)\frac{q-1}{2\mathrm{qN}}}F_{k}$$ (10)

Equation 10 tells us that the phase shift, depending on the diffraction order and *k*, should be additionally considered when we want to extend *F_k_* to the higher diffraction orders. Moreover, we can calculate an arbitrary *F*′_*k*′_ depending on the pixel size by slightly modifying the envelope function to a pixel size considered function, *ℰ*(*q*, *k*′) = *q* · sinc (π*k*′*p*/*Np*_0_), where *p* is the pixel size and *p*_0_ is the pixel pitch. We confirmed that the approximate sub-pixel consideration is same with the aforementioned emulating method within 0.3%.

### DAOH synthesis method details

Although DAOH without an additional phase can be synthesized using phase retrieval algorithms, DAOH with the additional phase cannot be synthesized with the methods. The primary obstacle arose when the constant-phase constraint applied at the SLM plane was transformed into correlated constraints involving both amplitude and phase (*U* = *e*^*i*ϕ(|*U*|^2^)^|*U*|). We attempted a two-step constraint to implement the correlated constraint: first, removing the phase of the field, and, then, applying additional phase modulation. However, the approach failed to converge, and, thus, we adopted a gradient descent method. We used automatic differentiation in PyTorch framework to implement the gradient descent optimization of DAOH. An Adam optimizer with a learning rate 0.002 is used to update the hologram by the gradient.

### Effect of sub-pixel induced noise

It is possible to present sub-pixel induced noise is relatively small in amplitude-only holograms by using TIE, $\frac{\partial }{\partial z}I\left(x,y,z\right)=\frac{\lambda }{2\pi }\nabla_{x,y}\cdot \left[I(x,y,z)\nabla_{x,y}\Phi (x,y,z)\right]$ where *I*(*x*, *y*, *z*) is intensity of light and Φ(*x*, *y*, *z*) is phase of light (*46*). Considering that sub-pixel noise originates from the difference between an ideal SLM and sub-pixel considered SLM, it is possible to write down the difference as

$$\begin{array}{c} \frac{\lambda }{2\pi }\nabla \cdot \left[I\nabla \Phi \right]-\frac{\lambda }{2\pi }\nabla \cdot \left[I\Theta (x,y)\nabla \Phi \right]\\ =\frac{\lambda }{2\pi }\nabla \cdot \left[I(1-\Theta (x,y))\nabla \Phi \right] \end{array}$$ (11)

where Θ(*x*, *y*) is a multiplied function of Heaviside step functions for the sub-pixel structure and *x*, *y*, and *z* are omitted in TIE. Equation 11 tells us that the sub-pixel noise can be minimized when the gradient of phase ∇Φ is zero. An ideal amplitude-only SLMs have zero phase gradient, and, thus, the sub-pixel noise is minimized. Although amplitude-only SLMs with phase noise have a non-zero phase gradient term, the phase gradient term would be much smaller than phase-only holograms.

Because TIE is the first-order approximation result with respect to *z*, someone may think that higher-order terms may change the conclusion. However, at least for the conditions used in Fig. 3 (A and B), we can confirm that amplitude-only holograms suffer less sub-pixel noise compared to phase-only holograms as inferred from Eq. 11. Moreover, as the propagation distance gets shorter, speckles induced by the sub-pixel structure becomes larger. As a result, the difference in image quality of Fig. 3 (A and B) also increases (see the Supplementary Materials for further details).

### Experimental setup

**Fig. 7.**
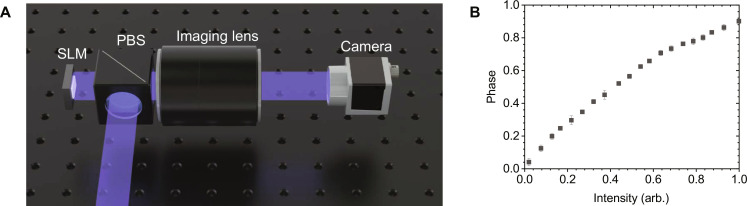
Experimental setup and measured phase noise. (**A**) Schematics of our experimental setup. The RGB lasers (blue) are shone on the SLM through the polarizing beam splitter (PBS). Reconstructed holograms are imaged by the imaging lens, and the translation stage moves camera back and forth to change the focus. (**B**) Measured additional phase versus intensity. As the intensity increases, additional phase also increases. Additional phases of the three colors are nearly the same, and, thus, the average data are plotted. The error bars represent the SDs between measurements.

Compared to other experimental setups for holographic displays, our experimental setup is relatively simple. Because optical filtering is not required in our system, LCoS with a pixel pitch of 7.2 μm, a polarizing beam splitter (PBS), camera, and imaging lens compose the entire experimental setup (Fig. 7A). IRIS-U62 (MAY Inc.) with 3840 × 2160 resolution and 3.6-μm pixel pitch is used as 1080p mode by putting same pixel value in 2 × 2 nearest pixels to minimize pixel cross-talk originating from its small pixel pitch (*47*). Collimated lasers with wavelengths of 638, 515, and 460 nm are illuminated through the PBS, and only modulated light goes through the PBS. The modulation intensity was calibrated by assigning one value to the whole pixels and measuring the modulated intensity.

### Measured phase noise of SLM

Measurement of the phase noise follows the method of the previous research (*35*). After beating two different wavefield generated by the same SLM, the relative phase is extracted by fitting the beating signal. Figure 7B shows the measured phase noise depending on intensities. Although higher order of polynomial fitting is expected to give a better result, we used first-order polynomial fitting in our experiment because it gives us reasonable results.

### Definitions of the metrics

To calculate the PSNR, we compared the numerically (optically) reconstructed intensity and the target intensity with the following formula

$$\text{PSNR}\equiv 10\text{log}\left(\frac{\text{Max}\left(I_{T}\right)}{\frac{1}{\mathrm{nm}}\sum_{j=0}^{m-1}‍\sum_{i=0}^{n-1}‍{\left|I_{R}\left(i,j\right)-I_{T}(i,j)\right|}^{2}}\right)$$ (12)

Here, *i* (*j*) represents the pixel index along the *x* (*y*) axis, *I*_R_(*i*, *j*) is the reconstructed intensity, and *I*_T_(*i*, *j*) is the target intensity. Because the field is clipped from 0 to 1, the maximum values of both intensities are always less than or equal to 1. The PSNR is defined on a logarithmic scale; a 3-dB difference in PSNR corresponds to twice the PSNR.

To calculate the SSIM, we compared the reconstructed intensity and the target intensity with the following formula

$$\begin{array}{c} \text{SSIM}\equiv \frac{1}{\mathrm{nm}}\sum_{j=0}^{m-1}‍\sum_{i=0}^{n-1}‍\frac{\left(2\mu_{R}(i,j)\mu_{T}(i,j)+c_{1}\right)}{\left(\mu_{R}^{2}(i,j)+\mu_{T}^{2}(i,j)+c_{1}\right)}\\ \times \frac{\left(2\sigma_{\text{RT}}\left(i,j\right)+c_{2}\right)}{\left(\sigma_{R}^{2}(i,j)+\sigma_{T}^{2}(i,j)+c_{2}\right)} \end{array}$$ (13)

where μ_R_(*i*, *j*) (μ_T_(*i*, *j*)) is the mean value of reconstructed (target) intensity with a window size *N*, $\sigma_{R}^{2}(i,j)\;\left(\sigma_{T}^{2}(i,j)\right)$ is the variance of reconstructed (target) intensity with a window size *N*, σ_RT_(*i*, *j*) is the covariance between the reconstructed and target intensity with a window size *N*, and *c*_1_ (*c*_2_) is the stabilization constant. In this work, we used *N* = 11, *c*_1_ = 0.0001, and *c*_2_ = 0.0009, which are the typically accepted values. In contrast to PSNR, SSIM is considered more sensitive to perceptual quality differences, such as textures, and the maximum value is limited to 1.

Last, the optical efficiency is defined as

$$\text{EFFI}\equiv \frac{1}{\mathrm{nm}}\sum_{j=0}^{m-1}‍\sum_{i=0}^{n-1}‍I_{R}(i,j)$$ (14)

Because the intensity is always less than or equal to 1, optical efficiency is always less than or equal to 1. The optical efficiency represents the ratio between the energy of the incident light and the energy of the reconstructed intensity.

### Effects of phase modulation on a twin image

To analyze the effect of phase modulation on a twin image, we synthesize and numerically reconstruct the DAOH with varying amounts of phase modulation. According to the “Derivation of phase noise of amplitude-only SLMs” section in the Supplementary Materials, the phase noise in amplitude-only SLMs is theoretically proportional to intensity. Thus, we maintained the relationship that the additional phase modulation is proportional to intensity while varying the proportional constant.

Specifically, we varied α in the following equation

$$U_{\text{SLM}}=e^{i\alpha {\left|U\right|}^{2}}\left|U\right|$$ (15)

For the value α = 1, the formula aligns with the experimental conditions and is used in the numerical simulation. After synthesizing the DAOH to reconstruct the target image at the plane *z* = *d*, we numerically reconstruct the DAOH at the *z* = *d* plane and the *z* = −*d* plane. Without additional phase modulation (α = 0), the reconstructed image at both planes would be the same, and the difference would get larger as α increases.

**Fig. 8.**
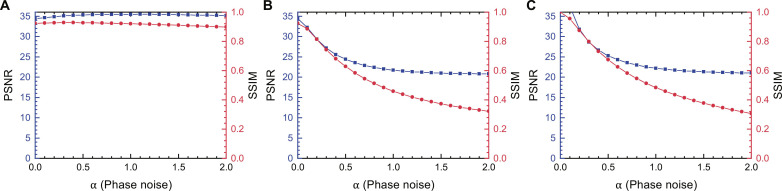
Image quality metrics of DAOH with varying the phase modulation. Average image quality metrics for DAOHs reconstructed at the target plane (**A**) and the conjugate plane (**B**). The image quality metrics are calculated between the target image and the reconstructed image. (**C**) Average image quality metrics between the reconstructed images at the target plane and the conjugate plane. Because the PSNRs and SSIMs are calculated between the two reconstructed images, the PSNR at α = 0 is infinite, and, thus, we do not plot the point in the figure. Blue squares represent PSNR, and red circles represent SSIM.

After synthesizing and reconstructing 100 images in the DIV2K validation dataset for different α values, we obtained Fig. 8. The image quality metrics of the reconstructed DAOHs at the target plane are nearly the same for different α values (Fig. 8A). In contrast, image quality metrics of the conjugate images rapidly decrease with increasing α (Fig. 8B). Figure 8C presents the image quality metrics between the reconstructed images on the target plane and the conjugate plane, indicating the similarities between them.

It is difficult to definitively identify the α value where the twin image disappears, but we can find the boundary where the two images become perceptually distinct. Because one would typically expect a substantial difference between any image and a plain white image, we set the reference SSIM as the SSIM between an image and a plain white image. More specifically, for each grayscale image in the same dataset used in Fig. 8, we calculated the SSIM between the image and a plain white image and averaged the results, leading to a reference SSIM of 0.46.

From Fig. 8B, it is possible to find the α value where the corresponding SSIM is equal to the reference SSIM, which is 0.46. Because Fig. 8B presents the SSIM between the conjugate image and the target image, the found α value corresponds to the point where twin image perceptually diminishes perfectly. The SSIM reaches 0.46 when α = 1.0, which is the same as that in the experimental condition.

## Supplementary Materials

### This PDF file includes:

Supplementary Text Figs. S1 to S12 References
