# Supplementary material for: Direct amplitude-only hologram realized by broken symmetry
Source: Sci Adv. 2024 Aug 30;10(35):eadp1205. doi: 10.1126/sciadv.adp1205 (PMC11364108; doi:10.1126/sciadv.adp1205)
Supplement: Supplementary file 1 — Supplementary Text Figs. S1 to S12 References [file sciadv.adp1205_sm.pdf]

Science Advances
AAAS

Supplementary Materials for

Direct amplitude-only hologram realized by broken symmetry

Daeho Yang and Hong-Seok Lee

Corresponding author: Daeho Yang, sheepvs5@gachon.ac.kr; Hong-Seok Lee, lhs12100@pknu.ac.kr

Sci. Adv. **10**, eadp1205 (2024)
DOI: 10.1126/sciadv.adp1205

This PDF file includes:

Supplementary Text
Figs. S1 to S12
References

Supplementary material

Effect of symmetry breaking by phase modulation

Since the derivation of Eq. M2 requires the condition of Eq. 1, applying Eq. 4 breaks the conjugate symmetry. Although it is not possible to derive a generalized relation between $U(x', y', z = d)$ and $U(x', y', z = -d)$ under an additional phase modulation, we can understand the relation by observing the representative example, a Gaussian beam. Assuming $|U(x, y, z = 0)| = e^{-\frac{x^2+y^2}{w_0^2}}$, wavefield of the Gaussian beam at the $z = 0$ plane, DAOH without additional phase modulation reconstructs the Gaussian beam with proper phase.

In contrast, assuming that additional phase modulation is given as a linear function of intensity, the wave function generated by the amplitude-only SLM would be given as,

$$U(x, y, z = 0) = e^{-\frac{x^2+y^2}{w_0^2}} e^{i\alpha e^{-2\frac{x^2+y^2}{w_0^2}}}, \tag{S1}$$

where α is a linear coefficient of the additional phase modulation. Under the condition $w_0 \gg \lambda$, which is valid for most of the cases, wavefront curvature $(R(z))$ of the Gaussian beam is much larger than the waist $(w(z))$ of the beam. Since the phase at near-zero intensity has little effect on the propagation of the wave, it is possible to approximate the wavefield with the additional phase factor as,

$$\begin{aligned} U(x, y, z = 0) &\approx e^{-\frac{x^2+y^2}{w_0^2}} e^{i\alpha - 2i\alpha\frac{x^2+y^2}{w_0^2} + i\alpha\mathcal{O}\left(\left(\frac{x^2+y^2}{w_0^2}\right)^2\right)} \\ &\equiv e^{i\alpha} e^{-\frac{x^2+y^2}{w'^2(d)}} e^{-ik\frac{x^2+y^2}{2R'(d)}}. \end{aligned} \tag{S2}$$

Here, $w'(d) = w_0$ and $R'(d) = kw_0^2/4\alpha$. The last term $e^{i\alpha\mathcal{O}\left(\left(\frac{x^2+y^2}{w_0^2}\right)^2\right)}$ becomes identity when $\sqrt{x^2+y^2} < w_0$ and becomes other values when $\sqrt{x^2+y^2} > w_0$. However, intensities of the field at $\sqrt{x^2+y^2} > w_0$ is nearly zero, the residual phase factor $(\mathcal{O}\cdots)$ does not influence much on wave propagation[46].

If we consider $U(x, y, z = 0)$ as a new Gaussian beam, it is possible to calculate modified waist (w_0') and modified center position (d) as a function of other parameters by using properties of the Gaussian beam. By using the equations, $w'(d) = w_0'\sqrt{1 + \left(\frac{2d}{kw_0'^2}\right)^2}$ and $R'(d) = d\left[1 + \left(\frac{kw_0'^2}{2d}\right)^2\right]$, simple algebra shows the relation,

$$\begin{aligned} w_0' &= \frac{w_0}{\sqrt{4\alpha^2+1}} \\ d &= \frac{\alpha}{4\alpha^2+1} k w_0^2. \end{aligned} \tag{S3}$$

For typical values of our experimental setup, *e.g.* $\alpha \approx 1$, the Gaussian beam with the reduced spot size is formed at a new plane, a few mm away from the SLM plane. In a Maxwellian view, defocus blur was much stronger when the focal plane was behind the SLM plane than the case of the focal plane was in front of the SLM plane (Fig. S1). Such asymmetric defocus blur can be understood in terms of Eq. S3. However, since Eq. S2 is different from an exact Gaussian beam, Eq. S3 should only be used to understand approximate tendency under the additional phase noise.

Optical reconstruction of conjugate image

Not only did we theoretically derive that the phase noise can eliminate the conjugate image, we also experimentally captured the conjugate plane to confirm nonexistence of the conjugate image. Figure S2 presents optically reconstructed DAOHs at the target plane and at the conjugate plane. PSNR differences between two planes are approximately 5 dB and the differences are sufficient for discriminating the focal plane. If the conjugate images are the same with the target images, then the differences should be much smaller than the current differences.

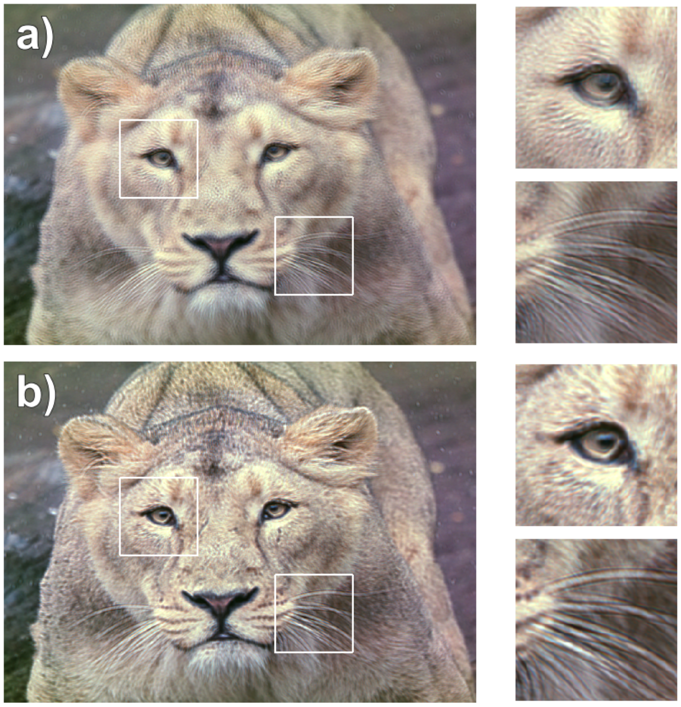

FIG. S1: **Experimental results of asymmetric defocus blur of Maxwellian display.** Captured images when the camera focus is backside(**a**) and frontside(**b**) of the SLM while a 2D image is displayed on the SLM. Photo Credit: Marcel Langthim.

Image quality of the phase-only hologram depending on the propagation distance

In the manuscript, the phase-only hologram is synthesized by the SGD algorithm and we used 250 mm distance to reconstruct the hologram. To clearly present image quality depending on the propagation distance, we simulated the reconstructed each result. In Fig. S3, image quality of the phase-only hologram is degraded by the sub-pixel structure and the reduced numerical aperture.

When the propagation distance is not enough, sub-pixel structure majorly degrades image quality. The sub-pixel structure induced noise arises from the fact that diffraction pattern reconstructed by a real SLM is different from that of the model used in optimization. Accordingly, the sub-pixel noise only influences on outer area of frequency domain, near the spatial frequency of the sub-pixel structure. As a result, propagating long enough distances can blow away the sub-pixel noise because high-spatial frequency components spread out rapidly.

In contrast, if the propagation distance is too long, then the spatial frequency bandwidth is limited by the reduced numerical aperture (Fig. S4). A tightly focused spot can be reconstructed by playing the point spread function (PSF) on the SLM. As the propagation distance gets longer, the total area of the PSF also gets larger. If the area of the PSF becomes larger than the area of the SLM, then it is not possible to reconstruct the sharply focused spot (Fig. S4**b**). Therefore, the spatial frequency bandwidth is limited by the reduced numerical aperture of the hologram.

Figure S4**c** presents image quality of phase-only holograms depending on the propagation distances, where the target images are DIV2K training dataset images. As the propagation distance increases, the image quality increases and then decreases due to the sub-pixel noise and the reduced numerical aperture. In the manuscript, we used the 250 mm propagation distance, since numerically reconstructed images with 250 mm or longer distance do not present background artifacts induced by white background.

Image quality metrics of the double-phase encoding method depending on the spatial bandwidth

Since the image quality of the double-phase encoding method depends on the aperture size of the spatial filtering, the aperture size presenting the best PSNR is used. Figure S5 presents the numerically simulated PSNRs and SSIMs of the double-phase encoding method depending on the spatial bandwidth limited by the aperture. For the spatial

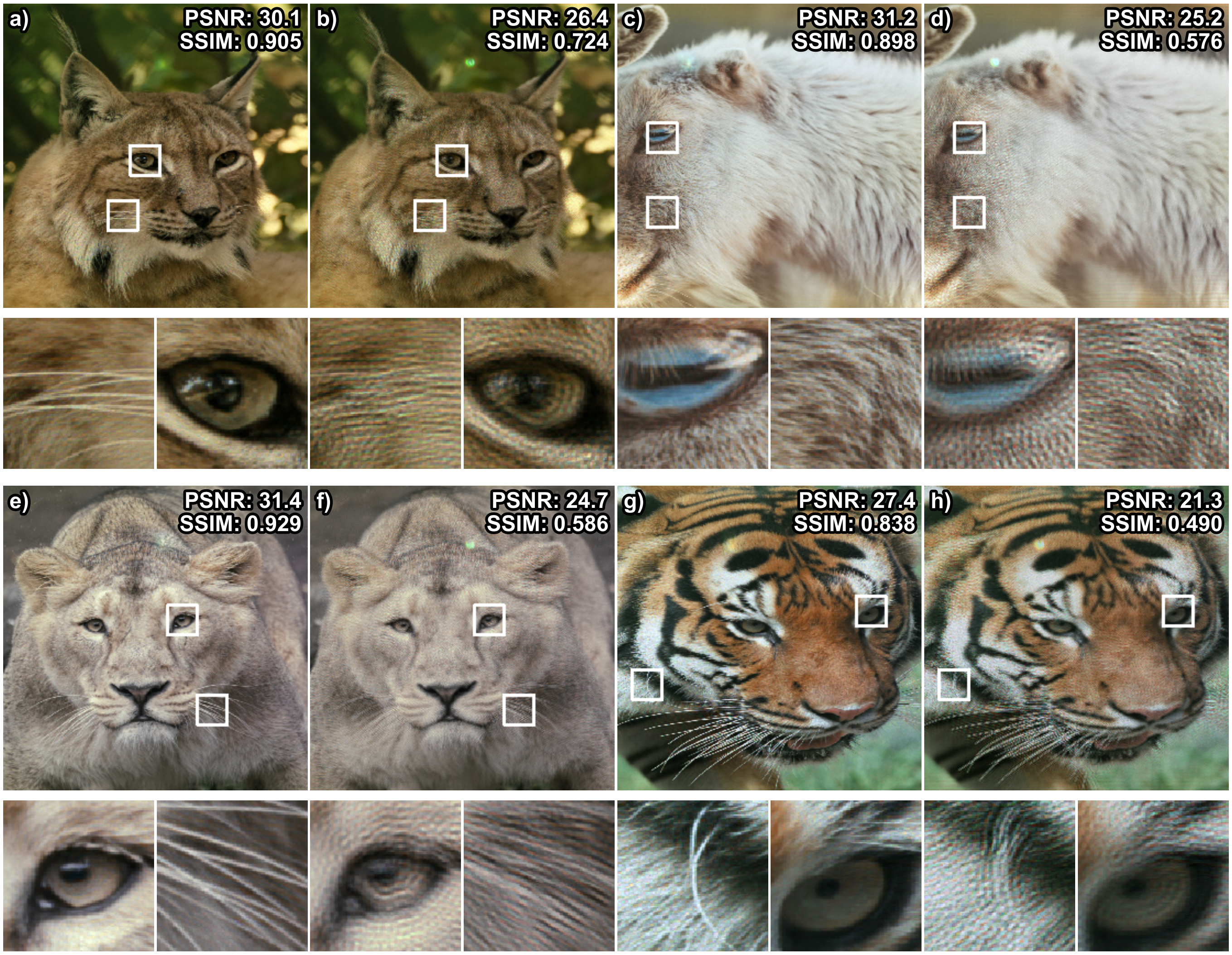

FIG. S2: **Optically reconstructed target and conjugate images. a, c, e, g,** Reconstructed DAOHs at the target plane (2 mm). **b, d, f, h,** Reconstructed DAOHs at the conjugate plane (-2 mm). Photo Credit: Greg Hume.

bandwidth 0.7, the method presents best image quality and thus the parameter is used in the study.

Realization with neural network

For a proof-of-concept, we trained a neural network to synthesize the DAOH (DAOHNet). We adopted the network architecture from the neural holography paper[34](Fig. S6), and used the loss function of Eq. 5. The DIV2K dataset[40] was used as a training dataset, where the images are resized to 944×1840. 40 pixels of padding were applied to all ends of the resized images, so the input shape of the network was 1024×1920. The neural network is trained for 300 epochs with a batch size 1.

Figure S7 presents the benchmark results of DAOHNet and other algorithms with the same condition. Since we used training dataset of DIV2K dataset while training, we used validation dataset of DIV2K dataset to benchmark the results. Image quality of DAOHNet is similar to DAOH and it is 700 times faster than DAOH. Moreover, image quality of DAOHNet is much better than that of the double-phase and Burch encoding methods.

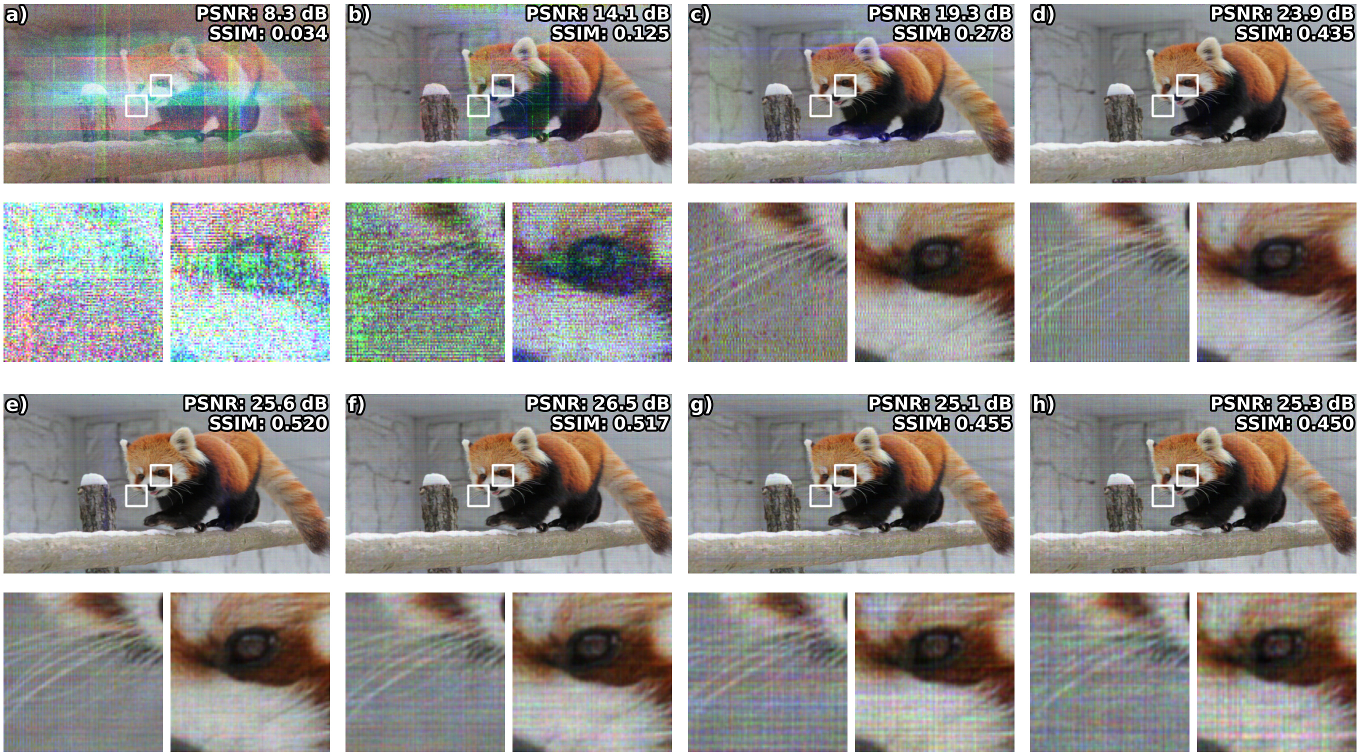

FIG. S3: **Image quality depending on the propagation distance for the phase-only holograms synthesized by the SGD algorithm.** Each propagation distance of the sub-figures corresponds to 50 mm, 100 mm, 150 mm, 200 mm, 250 mm, 300 mm, 350 mm, and 400 mm (from **a** to **h**). The image is selected from DIV2K dataset and resized to 1600×880. The edge padding with the shape, 160×100, is applied to the image, resulting the total image size 1920×1080[34]. The pixel pitch is assumed to 7.2 μm and the pixel size is assumed to 7.0 μm. Numerical reconstruction is modeled by ASM. Photo Credit: Lasermaster.

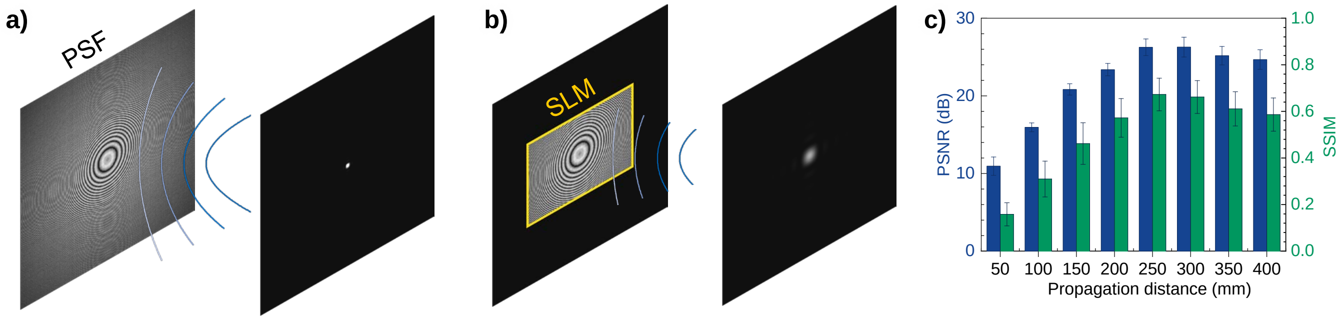

FIG. S4: **Reduced spatial frequency bandwidth of phase-only holograms a,** An ideal point spread function (PSF) can reconstruct a sharply focused spot. **b,** The PSF with restricted area by the SLM cannot reconstruct a sharply focused spot. **c,** Image quality metrics depending on the propagation distance. The image quality metrics are evaluated on DIV2K training dataset and the images are resized to 1920×1080 with the padding 160×100. The pixel pitch is assumed to 7.2 μm and the pixel size is assumed to 7.0 μm. Numerical reconstruction is modeled by ASM.

Experimental results with other target images

Figure S8 presents optically reconstructed intensities of the holograms. Experimental conditions are the same with the experiments in the manuscript except for the target intensities. All target intensities are images of the DIV2K training dataset[40].

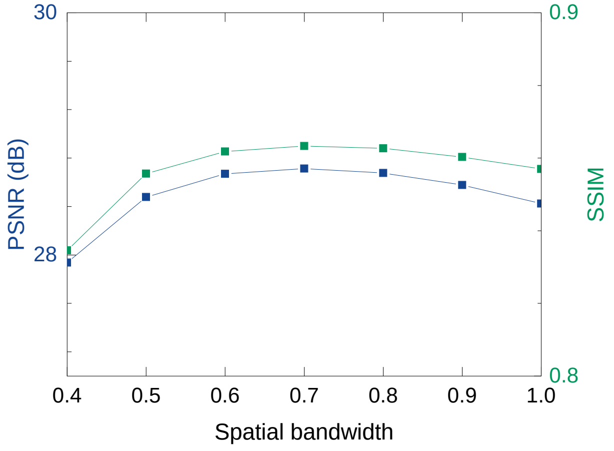

FIG. S5: **Image quality metrics of the double-phase encoding method depending on the spatial bandwidth.** Image quality metrics are evaluated on the DIV2K training dataset.

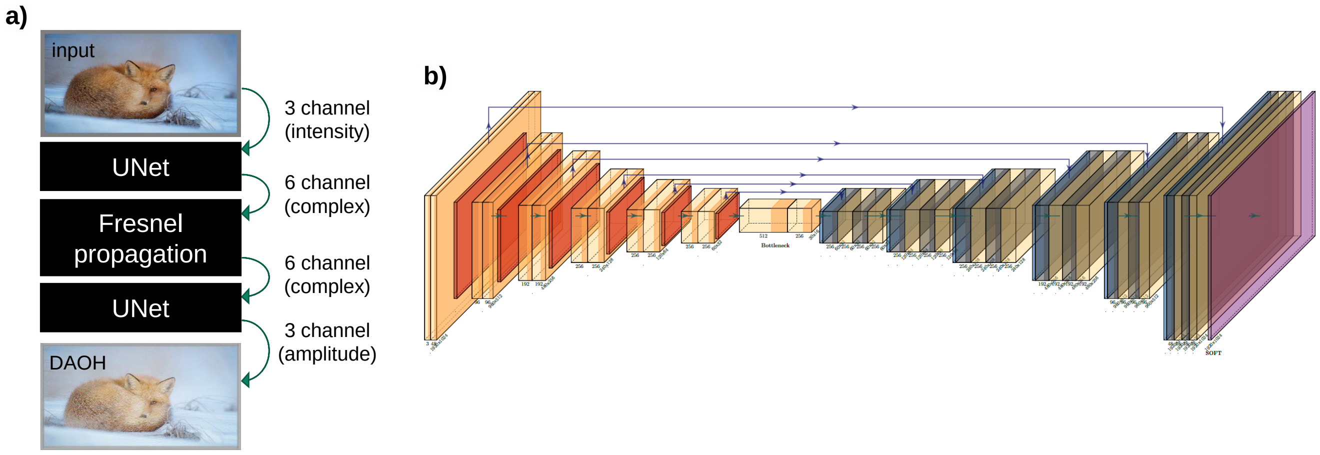

FIG. S6: **Schematics of neural network for DAOH. a,** Modules of the neural network for DAOH. The neural network for DAOH is composed of two UNets and a Fresnel propagator module. **b,** The structure of the UNet. Photo Credit: Ray Hennessy.

Derivation of phase noise of amplitude-only SLMs

The Jones matrix of vertical alignment LCoS is given by[47]

$$W = \begin{bmatrix} e^{-i\beta}cos\left(\beta\right) & -ie^{-i\beta}sin\left(\beta\right) \\ -ie^{-i\beta}sin\left(\beta\right) & e^{-i\beta}cos\left(\beta\right) \end{bmatrix}, \tag{S4}$$

where β is the birefringence. Here, the absolute phase is not neglected to explicitly derive the phase noise. For the linear polarization input $[1,0]^T$, the output field would be,

$$W\begin{bmatrix}1\\0\end{bmatrix} = \begin{bmatrix} e^{-i\beta}cos\left(\beta\right) \\ -ie^{-i\beta}sin\left(\beta\right) \end{bmatrix}. \tag{S5}$$

After a linear polarizer, only $e^{-i\beta}cos\left(\beta\right)$ term remains and the relation between the intensity and the phase can be calculated.

By expressing the relative intensity I_r compared to the input intensity 1, the additional phase can be derived as,

$$\phi\left(I_r\right) = -arccos\left(\sqrt{I_r}\right). \tag{S6}$$

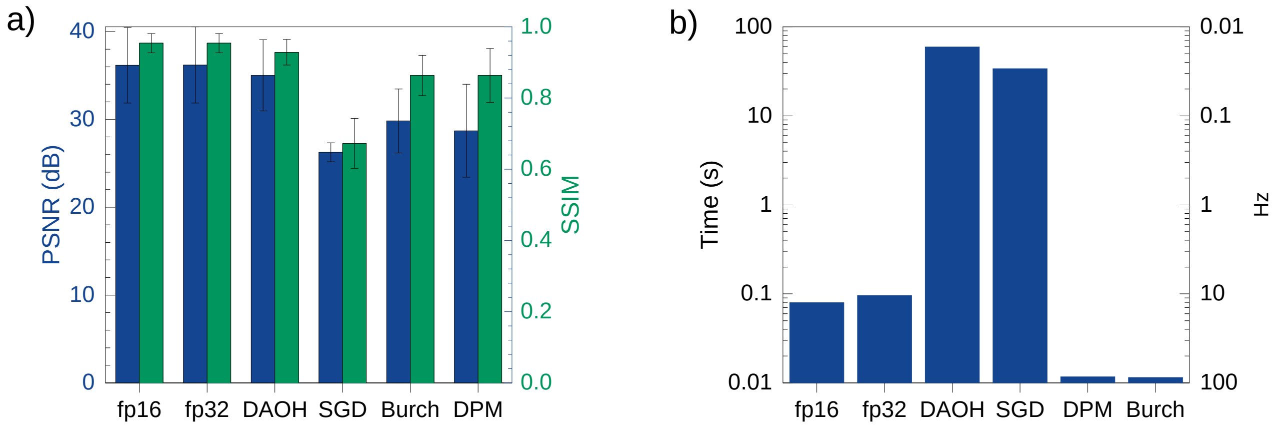

FIG. S7: **Benchmark results of the holograms including DAOHNet. a,** Image quality benchmark of the holograms. fp16 refers to DAOHNet with floating point 16 quantization and fp32 refers to DAOHNet without quantization. **b,** Hologram synthesis speed benchmark. Error bar represents standard deviation between the holograms with different target images.

As a result, unwanted phase modulation purely depends on the intensity, and it can be approximated as a linear function. Considering that the LCoS used in the experiment was vertical alignment LCoS, the result seems reasonable.

Demonstration of reconstructing a few points

To demonstrate an extreme case, we numerically reconstructed two points at different depths (Fig. S10). In the hologram, one point is located at the SLM plane and the other point is located at the floating plane where the distance between the SLM and floating plane is 2 mm. The reconstruction of both points was successful at each plane and the ringing patterns at defocus planes can be observed in the insets. In the insets, the intensity is enhanced tenfold without changing the spatial magnification. The ringing patterns of two points are slightly different due to the phase noise of the SLM.

Enhancing defocus blur of DAOH

Defocus blur can be limited due to the restricted scattering angle in non-diffusive holograms[35]. Since DAOH does not include loss term to increase defocus blur, the depth cue performance is expected to be not better than other non-diffusive holograms. However, by adopting proper loss functions, depth cue performance can be enhanced in DAOH. For proof-of-concept, gradient magnitude of intensity is adopted in the loss term to enhance defocus blur and the term can be written as,

$$\mathcal{L} = |\nabla I_G| \,. \tag{S7}$$

where, I_G refers to the intensity after applying gaussian blur. When the gradient magnitude loss is adopted, the image quality metrics are slightly decreased while the defocus blur is enhanced at other planes. Figure S11 presents defocus blur enhancement due to the gradient magnitude loss.

Optimization considering the sub-pixel structure

Under the perfect consideration of the sub-pixel structure, image quality can be further enhanced by including the sub-pixel structure in the optimization. However, the subpixel structure includes not only the fill-factor but also the fringe-field effect of the liquid crystal for liquid-crystal based SLMs. Consideration of the fringe-field effect is extensively hard since the fringe-field effect highly depends on the types of the liquid crystal, driving voltage, and the thickness of the liquid crystal[48]. Despite the non-perfect consideration of the sub-pixel structure, we attempted

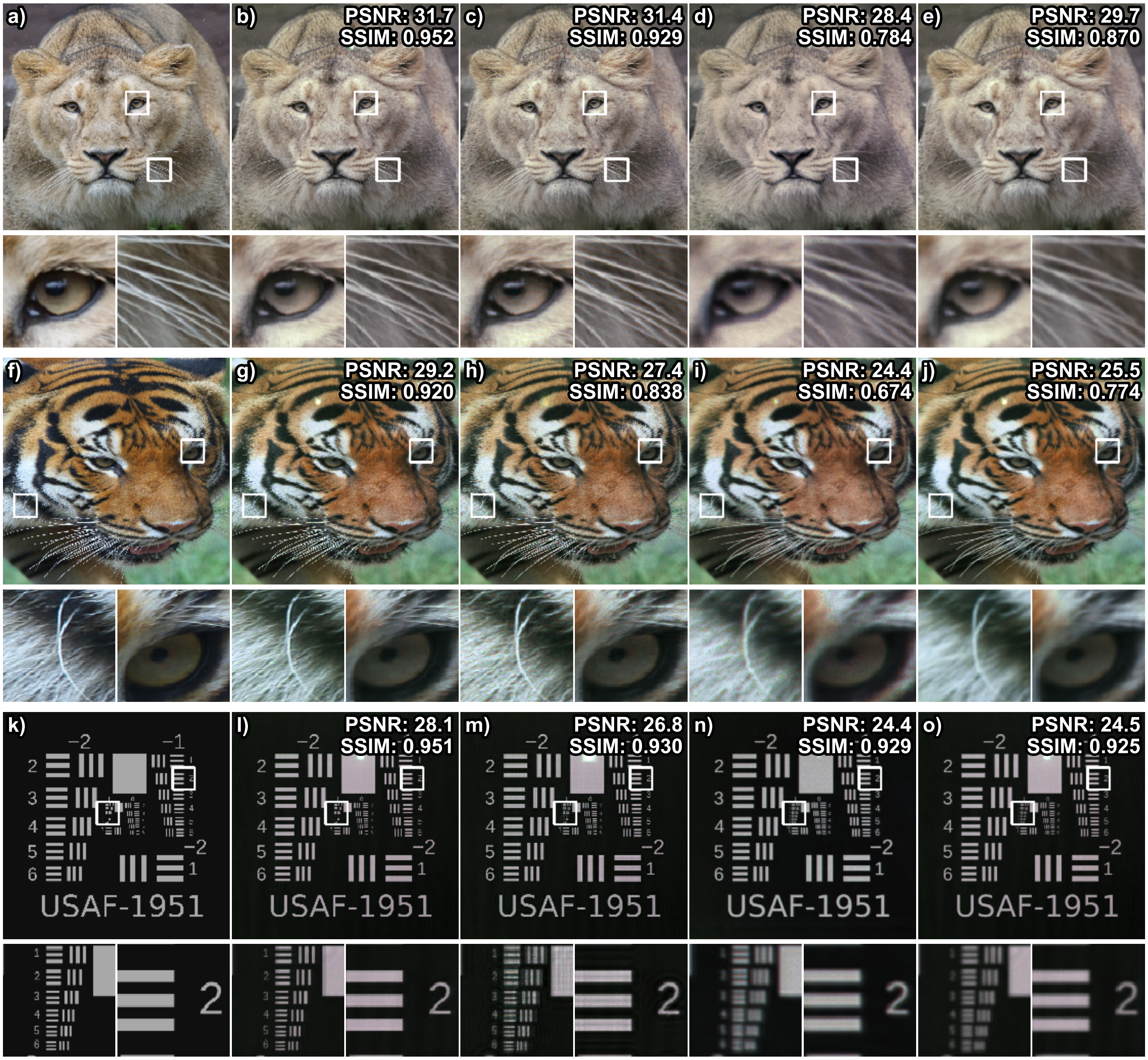

FIG. S8: **Experimental reconstruction of the holograms.** Target images (**a, f, k**), experimental-setup-limited images (**b, g, l**), optically reconstructed images of DAOHs (**c, h, m**), complex holograms with Burch encoding (**d, i, n**), and spatial-frequency-bandwidth-limited 2D images (**e, j, o**). Image quality metrics compared to the target images are marked on top-right side of the images. Small images present enlarged images of the above images. Photo Credit: Greg Hume.

to improve image quality by including only the fill-factor in the optimization process. However, improvement on the experimental image quality was not observed while improvement on the numerical result was substantial. Recent research[26] reported that partial consideration of the noise may enhance image quality in phase-only holograms, but the enhancement may not be observable in DAOH due to its robustness against sub-pixel noise. As a result, we used the fill-factor simulation to check the artifacts and to demonstrate the robustness of DAOH, not to optimize the hologram in the study.

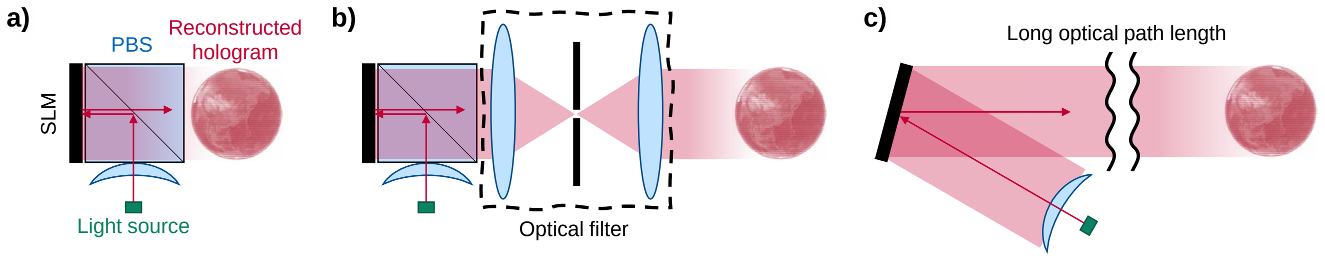

FIG. S9: **Schematic systems to reconstruct various holograms. a,** To reconstruct DAOHs, a light source and SLM are necessary. **b,** Complex holograms with encoding methods additionally require an optical filter. **c,** Phase-only holograms additionally require long optical path to sufficiently diffract light.

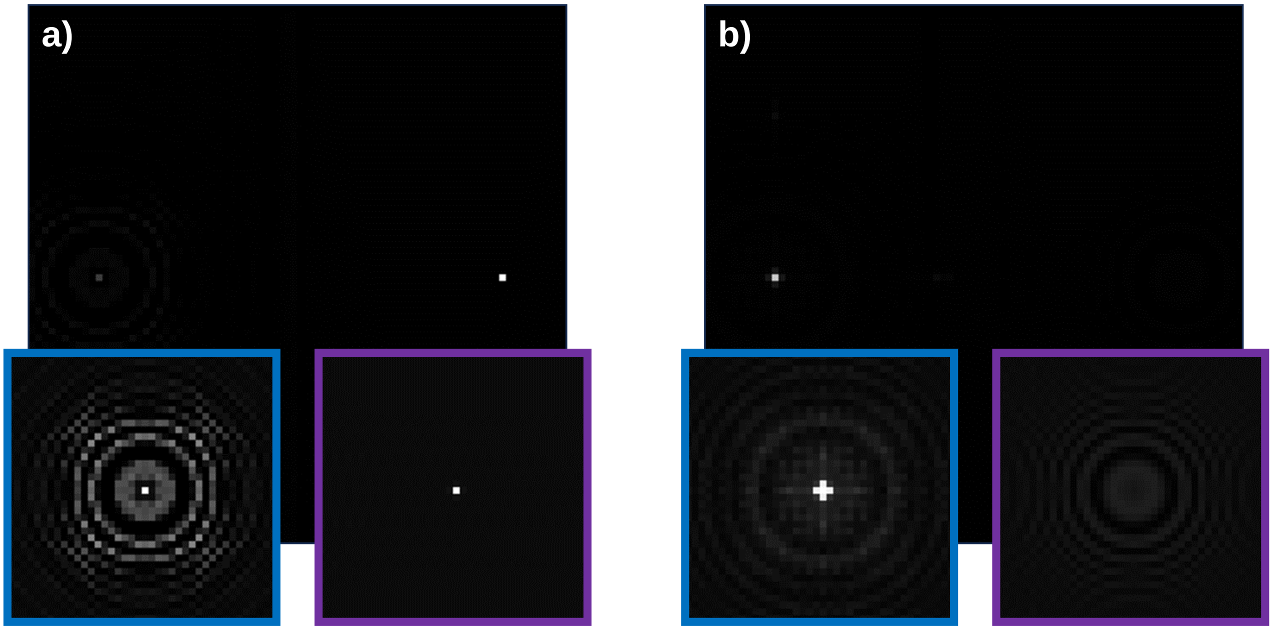

FIG. S10: **DAOH reconstructing two points floating at the different planes.** Numerically reconstructed image at the SLM plane (**a**) and at the other plane (**b**). The right point is at the SLM plane and the left point is at the 2mm floating plane from the SLM plane. To visualize the diffraction patterns, the insets present 10 times higher intensities without scale magnification.

Comparison with low-frequency images

We selected high-frequency images as target images in the manuscript to emphasize differences between holograms. In cases with fewer high-frequency components, these differences may be less substantial. To illustrate differences under low-frequency conditions, we utilized the Big Buck Bunny images as target images, which have also been employed in state-of-the-art phase-only hologram research[20, 34].

Figure S12 presents numerically reconstructed images of holograms for low-frequency targets. The visible image quality differences appear smaller compared to those in the manuscript because the PSNRs of reconstructed images are nearly 40 dB. Enlarged image magnifications are adjusted to a smaller value than that in the manuscript to maintain similar image qualities as the references.

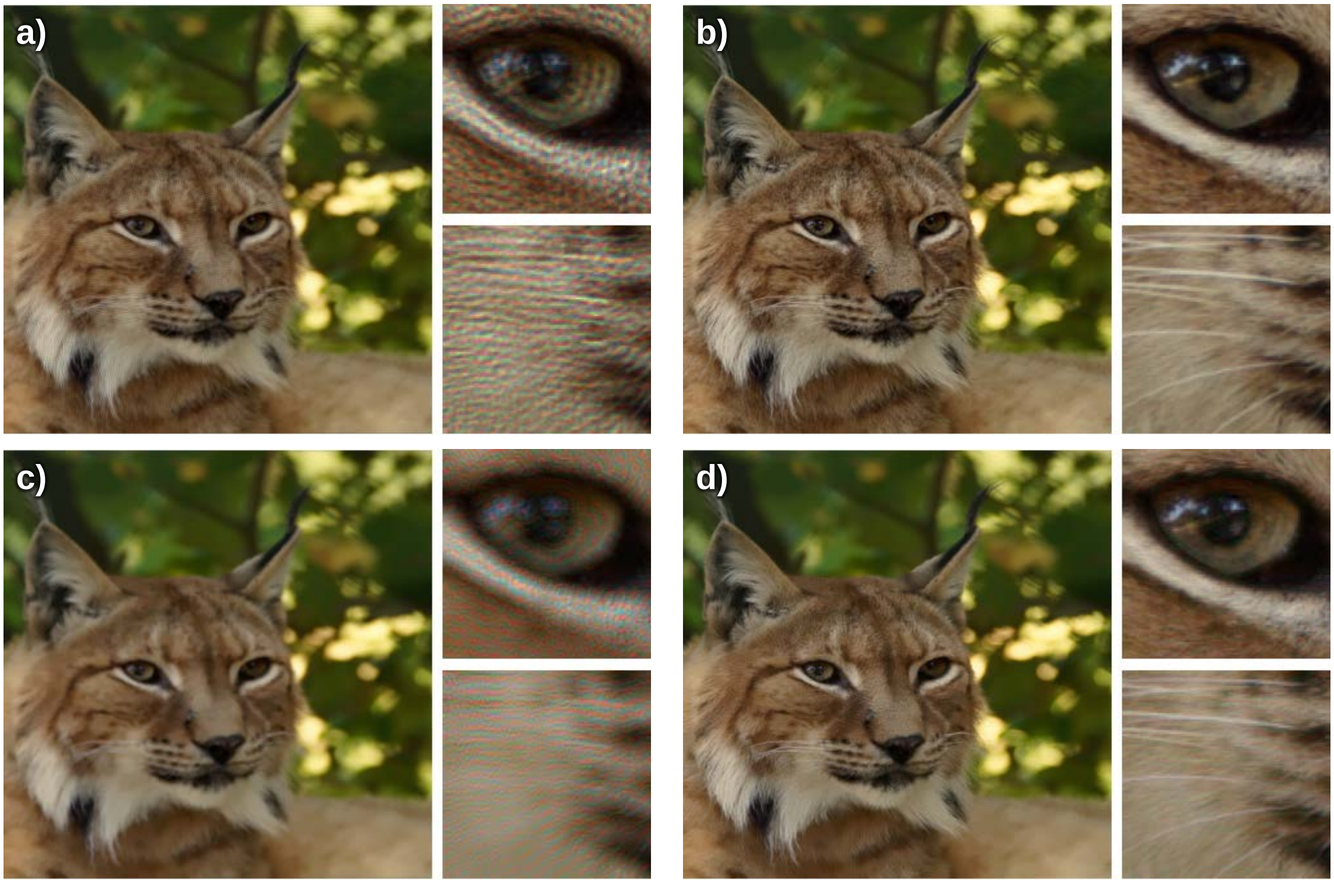

FIG. S11: **Effect of gradient magnitude loss in defocus blur.** Numerically reconstructed DAOH without (**a, b**) and with (**c, d**) the gradient magnitude loss term. The left images (right images) present numerically reconstructed images at z=-2 mm (z=2 mm) plane, where the reconstruction target plane is z=2 mm plane. Photo Credit: Ingo.

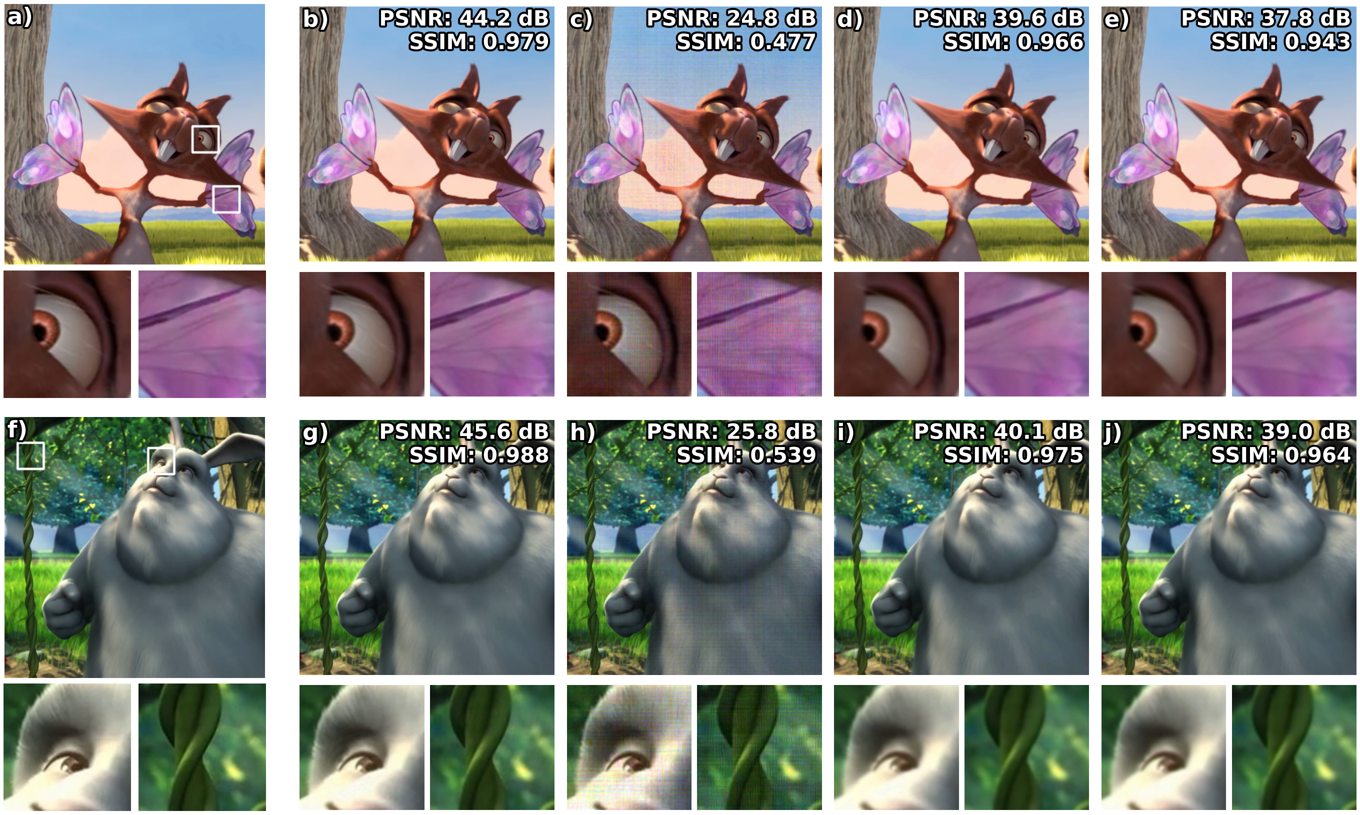

FIG. S12: **Numerically reconstructed holograms of low-frequency images.** Reconstruction target images (**a**, **f**). Reconstructed intensities of DAOHs (**b**, **g**), phase-only holograms (**c**, **h**), complex holograms with double-phase encoding (**d**, **i**), and complex holograms with Burch encoding (**e**, **j**) at the target plane. Peak signal-to-noise ratios (PSNRs) and structural similarity index measures (SSIMs) of reconstructed intensities compared to the target intensities are marked at the top-right side of the images. Photo Credit: Blender Foundation.

REFERENCES AND NOTES

1. D. Gabor, A new microscopic principle. *Nature* **161**, 777–778 (1948).

2. H. Yu, Y. Kim, D. Yang, W. Seo, Y. Kim, J.-Y. Hong, H. Song, G. Sung, Y. Sung, S.-W. Min, H.-S. Lee, Deep learning-based incoherent holographic camera enabling acquisition of real-world holograms for holographic streaming system. *Nat. Commun.* **14**,3534 (2023).

3. A. Vijayakumar, J. Rosen, Interferenceless coded aperture correlation holography–a new technique for recording incoherent digital holograms without two-wave interference. *Opt. Express* **25**, 13883–13896 (2017).

4. Y. K. Park, C. Depeursinge, G. Popescu, Quantitative phase imaging in biomedicine. *Nat. Photonics* **12**, 578–589 (2018).

5. J. Ashley, M.-P. Bernal, G. W. Burr, H. Coufal, H. Guenther, J. A. Hoffnagle, C. M. Jefferson, B. Marcus, R. M. Macfarlane, R. M. Shelby, G. T. Sincerbox, Holographic data storage technology. *IBM J. Res. Dev.* **44**, 341–368 (2000).

6. R. Erf, *Holographic Nondestructive Testing* (Elsevier, 2012).

7. W. Zhang, L. Cao, D. J. Brady, H. Zhang, J. Cang, H. Zhang, G. Jin, Twin-image-free holography: A compressive sensing approach. *Phys. Rev. Lett.* **121**, 093902 (2018).

8. T. Latychevskaia, H.-W. Fink, Solution to the twin image problem in holography. *Phys. Rev. Lett.* **98**, 233901 (2007).

9. Y. Pan, J. Liu, X. Li, Y. Wang, A review of dynamic holographic three-dimensional display: Algorithms, devices, and systems. *IEEE Trans. Industr. Inform.* **12**, 1599–1610 (2015).

10. I. Yamaguchi, "Phase-shifting digital holography: Principles and applications" in *Digital Holography and Three-Dimensional Display: Principles and Applications* (Springer, 2006), pp. 145–171.

11. F. Yaraş, H. Kang, L. Onural, State of the art in holographic displays: A survey. *J. Disp. Technol.* **6**, 443–454 (2010).

12. V. Arrizón, G. Méndez, D. Sánchez-de La-Llave, Accurate encoding of arbitrary complex fields with amplitude-only liquid crystal spatial light modulators. *Opt. Express* **13**, 7913–7927 (2005).

13. J. An, B. Shin, C.-K. Lee, C.-S. Choi, C. Yoo, G. Sung, H. Kim, H. Song, J.-S. Chung, J. Seo, K. Won, S.-H. Lee, S. Kim, W. Seo, Y. Kim, Y. Kim, Y.-T. Kim, H.-S. Lee, D. Lee, "7-2: High-contrast encoding method for amplitude-only computer generated hologram" in *SID Symposium Digest of Technical Papers* (John Wiley & Sons, 2018), vol. 49, pp. 64–67.

14. J. J. Burch, A computer algorithm for the synthesis of spatial frequency filters. *Proc. IEEE* **55**, 599–601 (1967).

15. C.-K. Hsueh, A. A. Sawchuk, Computer-generated double-phase holograms. *Appl. Optics* **17**, 3874–3883 (1978).

16. V. Arrizón, Complex modulation with a twisted-nematic liquid-crystal spatial light modulator: Double-pixel approach. *Opt. Lett.* **28**, 1359–1361 (2003).

17. S. A. Goorden, J. Bertolotti, A. P. Mosk, Superpixel-based spatial amplitude and phase modulation using a digital micromirror device. *Opt. Express* **22**, 17999–18009 (2014).

18. X. Li, J. Liu, J. Jia, Y. Pan, Y. Wang, 3D dynamic holographic display by modulating complex amplitude experimentally. *Opt. Express* **21**, 20577–20587 (2013).

19. D. Pi, J. Liu, S. Yu, Speckleless color dynamic three-dimensional holographic display based on complex amplitude modulation. *Appl. Optics* **60**, 7844–7848 (2021).

20. L. Shi, B. Li, C. Kim, P. Kellnhofer, W. Matusik, Towards real-time photorealistic 3D holography with deep neural networks. *Nature* **591**, 234–239 (2021).

21. X. Sui, Z. He, G. Jin, D. Chu, L. Cao, Band-limited double-phase method for enhancing image sharpness in complex modulated computer-generated holograms. *Opt. Express* **29**, 2597–2612 (2021).

22. L. Shi, B. Li, W. Matusik, End-to-end learning of 3D phase-only holograms for holographic display. *Light Sci. Appl.* **11**, 247 (2022).

23. M. H. Maleki, A. J. Devaney, Phase-retrieval and intensity-only reconstruction algorithms for optical diffraction tomography. *J. Opt. Soc. Am. A* **10**, 1086–1092 (1993).

24. J. Park, K. R. Lee, Y. K. Park, Ultrathin wide-angle large-area digital 3D holographic display using a non-periodic photon sieve. *Nat. Commun.* **10**, 1304 (2019).

25. K. Bang, C. Jang, B. Lee, Compact noise-filtering volume gratings for holographic displays. *Opt. Lett.* **44**, 2133–2136 (2019).

26. M. Gopakumar, J. Kim, S. Choi, Y. Peng, G. Wetzstein, Unfiltered holography: Optimizing high diffraction orders without optical filtering for compact holographic displays. *Opt. Lett.* **46**, 5822–5825 (2021).

27. F. Yang, A. Kadis, R. Mouthaan, B. Wetherfield, A. Kaczorowski, T. D. Wilkinson, Perceptually motivated loss functions for computer generated holographic displays. *Sci. Rep.* **12**, 7709 (2022).

28. B. Lee, D. Kim, S. Lee, C. Chen, B. Lee, High-contrast, speckle-free, true 3D holography via binary CGH optimization. *Sci. Rep.* **12**, 2811 (2022).

29. Y. K. Kim, W. J. Ryu, J. S. Lee, Study of non-periodic pinhole array filter for decreasing high-order noise for compact holographic display. *Appl. Sci.* **10**, 8671 (2020).

30. R. W. Gerchberg, A practical algorithm for the determination of phase from image and diffraction plane pictures. *Optik* **35**, 237–246 (1972).

31. C. Chang, J. Xia, L. Yang, W. Lei, Z. Yang, J. Chen, Speckle-suppressed phase-only holographic three-dimensional display based on double-constraint Gerchberg–Saxton algorithm. *Appl. Optics* **54**, 6994–7001 (2015).

32. R. D. Juday, Correlation with a spatial light modulator having phase and amplitude cross coupling. *Appl. Optics* **28**, 4865–4869 (1989).

33. K. Matsushima, T. Shimobaba, Band-limited angular spectrum method for numerical simulation of free-space propagation in far and near fields. *Opt. Express* **17**, 19662–19673 (2009).

34. Y. Peng, S. Choi, N. Padmanaban, G. Wetzstein, Neural holography with camera-in-the-loop training. *ACM Trans. Graph.* **39**, 1–14 (2020).

35. D. Yang, W. Seo, H. Yu, S. I. Kim, B. Shin, C.-K. Lee, S. Moon, J. An, J.-Y. Hong, G. Sung, H.-S. Lee, Diffraction-engineered holography: Beyond the depth representation limit of holographic displays. *Nat. Commun.* **13**, 6012 (2022).

36. D. Kim, S.-W. Nam, B. Lee, J.-M. Seo, B. Lee, Accommodative holography: Improving accommodation response for perceptually realistic holographic displays. *ACM Trans. Graph.* **41**, 1–15 (2022).

37. D. Yoo, Y. Jo, S.-W. Nam, C. Chen, B. Lee, Optimization of computer-generated holograms featuring phase randomness control. *Opt. Lett.* **46**, 4769–4772 (2021).

38. S. Choi, M. Gopakumar, Y. Peng, J. Kim, M. O'Toole, G. Wetzstein, "Time-multiplexed neural holography: A flexible framework for holographic near-eye displays with fast heavily-quantized spatial light modulators" in *ACM SIGGRAPH 2022 Conference Proceedings* (ACM, 2022), pp. 1–9.

39. D. Kim, S.-W. Nam, S. Choi, J.-M. Seo, G. Wetzstein, Y. Jeong, Holographic parallax improves 3D perceptual realism. arXiv:2404.11810 (2024).

40. E. Agustsson, R. Timofte, "NTIRE 2017 challenge on single image super-resolution: Dataset and study" in *The IEEE Conference on Computer Vision and Pattern Recognition (CVPR) Workshops* (IEEE, 2017), pp. 126–135.

41. D. Yang, Enhancing efficiency of complex field encoding for amplitude-only spatial light modulator based on a neural network. *Opt. Express* **31**, 40741–40747 (2023).

42. H. Yang, D. P. Chu, Phase flicker in liquid crystal on silicon devices. *J. Phys. Photonics* **2**, 032001 (2020).

43. Y. Peng, S. Choi, J. Kim, G. Wetzstein, Speckle-free holography with partially coherent light sources and camera-in-the-loop calibration. *Sci. Adv.* **7**, eabg5040 (2021).

44. J. An, K. Won, Y. Kim, J.-Y. Hong, H. Kim, Y. Kim, H. Song, C. Choi, Y. Kim, J. Seo, A. Morozov, H. Park, S. Hong, S. Hwang, K. Kim, H.-S. Lee, Slim-panel holographic video display. *Nat. Commun.* **11**, 5568 (2020).

45. B. E. A. Saleh, M. C. Teich, *Fundamentals of Photonics* (John Wiley & Sons, 2019).

46. M. R. Teague, Deterministic phase retrieval: A green's function solution. *J. Opt. Soc. Am.* **73**, 1434–1441 (1983).

47. G. Lazarev, A. Hermerschmidt, S. Krüger, S. Osten, "LCOS spatial light modulators: Trends and applications" in *Optical Imaging and Metrology: Advanced Technologies* (John Wiley & Sons, 2012), pp. 1–29.

48. Y. Huang, E. Liao, R. Chen, S.-T. Wu, Liquid-crystal-on-silicon for augmented reality displays. *Appl. Sci.* **8**, 2366 (2018).
